# Diagnostic Accuracy of Artificial Intelligence and Computer-Aided Diagnosis for the Detection and Characterization of Colorectal Polyps: Systematic Review and Meta-analysis

**DOI:** 10.2196/27370

**Published:** 2021-07-14

**Authors:** Scarlet Nazarian, Ben Glover, Hutan Ashrafian, Ara Darzi, Julian Teare

**Affiliations:** 1 Department of Surgery and Cancer Imperial College London London United Kingdom

**Keywords:** artificial intelligence, colonoscopy, computer-aided diagnosis, machine learning, polyp

## Abstract

**Background:**

Colonoscopy reduces the incidence of colorectal cancer (CRC) by allowing detection and resection of neoplastic polyps. Evidence shows that many small polyps are missed on a single colonoscopy. There has been a successful adoption of artificial intelligence (AI) technologies to tackle the issues around missed polyps and as tools to increase the adenoma detection rate (ADR).

**Objective:**

The aim of this review was to examine the diagnostic accuracy of AI-based technologies in assessing colorectal polyps.

**Methods:**

A comprehensive literature search was undertaken using the databases of Embase, MEDLINE, and the Cochrane Library. PRISMA (Preferred Reporting Items for Systematic Reviews and Meta-Analyses) guidelines were followed. Studies reporting the use of computer-aided diagnosis for polyp detection or characterization during colonoscopy were included. Independent proportions and their differences were calculated and pooled through DerSimonian and Laird random-effects modeling.

**Results:**

A total of 48 studies were included. The meta-analysis showed a significant increase in pooled polyp detection rate in patients with the use of AI for polyp detection during colonoscopy compared with patients who had standard colonoscopy (odds ratio [OR] 1.75, 95% CI 1.56-1.96; *P*<.001). When comparing patients undergoing colonoscopy with the use of AI to those without, there was also a significant increase in ADR (OR 1.53, 95% CI 1.32-1.77; *P*<.001).

**Conclusions:**

With the aid of machine learning, there is potential to improve ADR and, consequently, reduce the incidence of CRC. The current generation of AI-based systems demonstrate impressive accuracy for the detection and characterization of colorectal polyps. However, this is an evolving field and before its adoption into a clinical setting, AI systems must prove worthy to patients and clinicians.

**Trial Registration:**

PROSPERO International Prospective Register of Systematic Reviews CRD42020169786; https://www.crd.york.ac.uk/prospero/display_record.php?ID=CRD42020169786

## Introduction

Colorectal cancer (CRC) is the third-leading malignancy worldwide and a leading cause of mortality [1]. CRC typically develops from sporadic colorectal adenomatous polyps, and colonoscopy is established for the detection and resection of these lesions, which has been shown to reduce the incidence and mortality from CRC [2]. However, as with any procedure, endoscopic polyp detection has operator-dependent limitations. There is evidence highlighting that small polyps may be missed at colonoscopy with a miss rate for adenomas as high as 26% [3]. The primary colonoscopy quality indicator is the adenoma detection rate (ADR). Given that ADR is inversely proportional to postcolonoscopy CRC risk, with each 1% increase in ADR equivalent to a 3% decrease in the subsequent risk of cancer [4], there is an unmet need to tackle the problems that prevent high-quality colonoscopy.

Human and technical factors lead to a small but significant proportion of missed polyps during colonoscopy. Several studies have suggested that ADR can be increased by improving the educational and behavioral skills of the endoscopist. Training programs, consisting of hands-on teaching and regular feedback, showed good results in increasing ADR in trials [5,6]. However, the increase in detection from baseline in these studies was minimal and the ability of even expert endoscopists to detect very small, subtle, or flat lesions remains a limiting factor.

Recently, there has been a successful adoption of artificial intelligence (AI) technologies in health care diagnostics [7]. The ability of AI, specifically machine learning approaches, to differentiate and characterize distinct pathologies is continuously enhancing early computer-aided diagnosis (CAD) techniques. Deep learning models are built using artificial neural networks and have proven very useful with analysis of big data in health care. Convolutional neural networks (CNNs) and their variants with AI models have become the most preferred and widely used methods in medical image analysis. Convolutional layers convolve the input and pass its result to the next layer. Application of AI in colonoscopy has focused more on polyp detection than characterization, driven by the development of deep CNNs (DCNNs). The architecture of these algorithms includes multiple layers of processing between the input and output layers, allowing analysis of complex data with efficient performance. The most advanced polyp detection systems are those that can be applied to video-based analysis during colonoscopy.

In the field of endoscopy, a machine learning algorithm can be trained to recognize or characterize polyps in real time. Two endoscopic approaches have been studied: techniques used for analysis of nonmagnified endoscopic images and those for cellular imaging at a microscopic level (ie, optical biopsy).

The idea of such approaches is that by detecting more polyps (ie, increasing the polyp detection rate [PDR]), there will be a corresponding reduction in the number of missed adenomas and, consequently, a reduction in the subsequent risk of CRC. However, this presents a financial burden on health care systems, especially the histopathology departments, involved in analysis of resected tissue, which will only increase with the increase in detection of polyps. The ultimate goal of a CAD system would be the reliable detection of every polyp within the colon during the colonoscopy procedure, while also characterizing them as hyperplastic or adenomatous to guide decision making for polypectomy and histopathological examination [8]. The Preservation and Incorporation of Valuable endoscopic Innovations (PIVI) initiative, set by the American Society of Gastrointestinal Endoscopy (ASGE), has established a desired threshold for the introduction of new endoscopic technologies, including the optical diagnosis of diminutive colorectal polyps [9]. Despite several, predominantly single-site, studies meeting the PIVI criteria showing that a “resect and discard” strategy or a “diagnose and leave” strategy could be adopted [10,11], a recent multicenter study showed that the accuracy of optical diagnosis requires imaging advances before it can be used to determine surveillance without histology [12].

Machine learning by definition is a model that is able to constantly adapt and improve when presented with new information. To ensure this refinement, large quantities of good-quality data should be used for training the algorithm. Current AI systems that are not synthesized in this way are prone to the risk of *overfitting*, whereby the system performs well with training data to the extent that it negatively impacts its performance when tested on new data [13]. Thus, for an AI system to be successful in its ability to detect and characterize polyps, it should adopt a machine learning model based on good-quality high-yield data and the model should have a high sensitivity for the detection of polyps, have a low rate of false positives, and be able to maintain fast processing speeds to be applicable in near-real time during colonoscopy [14].

Our aims were to systematically review and meta-analyze the diagnostic accuracy of AI-based technologies in the detection and characterization of colorectal polyps.

## Methods

This review was carried out and reported in accordance with the PRISMA (Preferred Reporting Items for Systematic Reviews and Meta-Analyses) statement [15]. It has been registered on PROSPERO (International Prospective Register of Systematic Reviews) (registration No. CRD42020169786).

### Search Strategy

A comprehensive literature search was undertaken using the databases of Embase, MEDLINE, and the Cochrane Library. All published articles up until October 2020 were included. Search terms used in Embase and MEDLINE included “colon*,” “polyp,” “artificial intelligence OR machine learning,” and “computer aided or assisted and diagnos* OR detect*.” Studies in the Cochrane Library were identified with the terms “colonic polyp,” “artificial intelligence,” and “diagnosis, computer-assisted” (Multimedia Appendix 1).

### Inclusion and Exclusion Criteria

Inclusion criteria were as follows:

- Studies reporting computer-aided detection of colorectal polyps retrospectively, using endoscopic images or videos
- Studies reporting computer-aided classification of colorectal polyps retrospectively, using endoscopic images or videos
- Studies reporting the use of CAD of colorectal polyps during colonoscopy
- Studies reporting ADR, PDR, sensitivity, specificity, and diagnostic accuracy data or studies with adequate information to calculate these data
- Studies published or translated into English.

Exclusion criteria were as follows:

- Studies with no original data present (eg, review article or letter)
- Studies with no full text available
- Studies conducted in patients with inflammatory bowel disease (IBD)
- Studies greater than 20 years old
- Studies without adequate data to calculate sensitivity, specificity, and diagnostic accuracy data; PDR and ADR; adenoma miss rate; or mean adenomas per patient, or those not reporting these data.

### Study Selection

The retrieved articles were screened for duplicates by two reviewers; these were excluded. Titles and abstracts were then screened for relevance by two reviewers independently, and irrelevant studies were excluded. Following this, full-text reviews of remaining studies were completed. The reference lists of identified review articles and included papers were scrutinized for relevant studies. Disagreements about eligibility were settled by consensus, both after screening and following full‐text review. Inclusion and exclusion criteria were met by all final articles.

### Data Extraction

Data were gathered from studies and placed onto a standard spreadsheet template. For each study, we extracted the following data: study details (ie, first author, year of publication, and journal), primary outcome (ie, polyp detection vs characterization), study design (ie, type of study, method of AI, and exclusion criteria), information on type of imaging modality (ie, images or videos, images for training, and images for validation), and information regarding diagnostic accuracy characteristics (ie, sensitivity, specificity, accuracy, ADR, and PDR).

### Study Quality Assessment

Study quality was independently assessed using the Quality Assessment of Diagnostic Accuracy Studies 2 (QUADAS-2) tool [16]. Each domain was classified as low-risk, high-risk, or unclear risk of bias. For randomized controlled trials (RCTs), the Jadad scale was used for quality scoring [17]. Studies with a Jadad score of 3 or more were considered *good* quality.

### Statistical Analysis

Independent proportions and their differences were calculated and pooled through DerSimonian and Laird random-effects modeling. This considered both between-study and within-study variances, which contributed to study weighting. Pooled values and 95% CIs were computed and represented on forest plots. Statistical heterogeneity was determined by the I^2^ statistic, where <30% was low, 30%-60% was moderate, and >60% was high. Analyses were performed using Stata, version 15 (StataCorp LLC). Probability values of *P*≤.05 were considered statistically significant.

## Results

### Search Results and Characteristics

**Figure 1:**
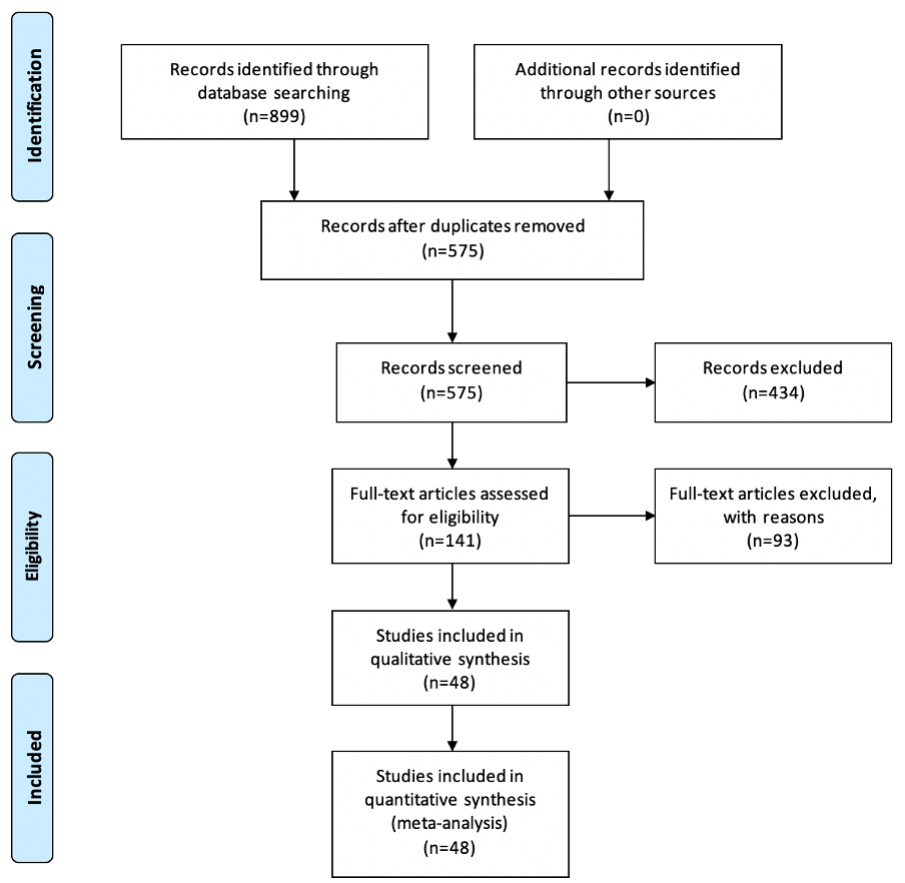
PRISMA (Preferred Reporting Items for Systematic Reviews and Meta-Analyses) flow diagram for study selection.

A total of 899 articles were identified from the database searches. After removing duplicates, 575 records were screened on the basis of titles and abstracts. A total of 141 articles were identified as appropriate for full-text review. Further evaluation and application of the exclusion criteria revealed 48 studies, which were included in this systematic review and meta-analysis. The study screening and selection process is shown in Figure 1.

Studies in this systematic review included preclinical studies for polyp detection (Table 1 [18-35]), preclinical studies for polyp characterization (Table 2 [11,13,36-55]), and recent RCTs (Table 3 [56-63]). The studies were all published between 2003 and 2020. The outcome measures were polyp detection in 18 studies, polyp characterization in 22 studies, and PDR in 8 studies. The studies analyzing sensitivity, specificity, and accuracy when testing each AI system were found to present results at the per-patient, per-polyp, and/or per-image levels, whereas the RCTs evaluating the ADR and PDR consistently presented per-patient results.

Studies for polyp detection predominantly used CNN or DCNN as their machine learning approach. A total of 14 studies for polyp detection were carried out retrospectively. There was a large variation in the number of images used by each paper to train or validate the AI systems in detecting polyps, with one study using 8 images [20] to train the system, while another used 5545 images [25].

In the majority of studies, narrow band imaging (NBI) or endocytoscopy was the imaging method of choice for characterizing polyps, with one exception in which the imaging modality was not stated [47]. Data for polyp characterization was gathered retrospectively in 18 studies. In 3 studies that collected data prospectively, a support vector machine classifier was used as the machine learning approach. Similarly to studies for polyp detection, those analyzing polyp characterization had a large variation in number of images used for training or validating the AI system. However, studies for polyp characterization focused more on the number of polyps used than on overall images, as seen in Table 2.

**Table 1:** Characteristics of included studies whose primary outcome was polyp detection.

Authors	Year	Recruitment	Machine learning approach	Imaging modality	Patients, n	Polyps, n	Total images, n	Images for training, n	Images for validation, n
Karkanis et al [18]	2003	Retrospective	CWC^a^	RGB^b^–color frame grabber	66	95	1380	180	1200
Fu et al [19]	2014	Retrospective	SFFS^c^ with SVM^d^ classifier	Still image enhanced by PCT^e^	100	—^f^	365	292	73
Wang et al [20]	2015	Retrospective	Polyp edge detection—ECSP^g^	Video clip	—	43	—	8	53
Tajbakhsh et al [21]	2015	Retrospective	Hybrid context-shape approach	CVC^h^-Colon database	—	15	300	300	—
Tajbakhsh et al [21]	2015	Retrospective	Hybrid context-shape approach	ASU^i^-Mayo database	—	10	19,400	300	—
Fernández-Esparrach et al [22]	2016	Retrospective	WM-DOVA^j^ maps	White light colonoscope	—	31	612	—	—
Park and Sargent [23]	2016	Retrospective	CNN^k^-CRF^l^ model	White light and NBI^m^	—	92	11,802	—	—
Urban et al [24]	2018	Retrospective	DCNN^n^	NBI images	>2000	—	8641	—	—
Wang et al [25]	2018	Retrospective	ANN^o^- SegNet architecture	Still images	2428	—	—	5545	27,113
Misawa et al [26]	2018	Retrospective	CNN	White light images	73	155	546	411	135
Figueiredo et al [27]	2019	Retrospective	SVM binary classifier	White light images	42	42	—	—	—
Yamada et al [28]	2019	Retrospective	Faster R-CNN^p^	Still images	—	752	—	>4000	4840
Becq et al [29]	2020	Prospective	ANN- SegNet architecture	Video	50	165	—	—	—
Gao et al [30]	2020	Retrospective	CNN	White light images	—	—	1709	1196	256
Guo et al [31]	2020	Retrospective	CNN-YOLO^q^	Video	283	—	1991	—	—
Lee et al [32]	2020	Prospective	CNN-YOLO	Video	15	26	—	8495	110,728
Ozawa et al [33]	2020	Retrospective	CNN	NBI and white light	12,895	309	—	20,431	7077
Misawa et al [34]	2020	Prospective	CNN-YOLO	White light images	1405	100	56,668	51,889	4769
Poon et al [35]	2020	Prospective	CNN-ResNet50, YOLO	Video	144	128	—	198,138	34,469

^a^CWC: color wavelet covariance.

^b^RGB: red, green, and blue.

^c^SFFS: sequential floating-forward selection.

^d^SVM: support vector machine.

^e^PCT: principal components transformation.

^f^This value was not reported.

^g^ECSP: edge cross-section profiles.

^h^CVC: Computer Vision Center.

^i^ASU: Arizona State University.

^j^WM-DOVA: window median depth of valleys accumulation.

^k^CNN: convolutional neural network.

^l^CRF: conditional random field.

^m^NBI: narrow band imaging.

^n^DCNN: deep convolutional neural network.

^o^ANN: artificial neural network.

^p^R-CNN: region-based convolutional neural network.

^q^YOLO: you only look once.

**Table 2:** Characteristics of included studies whose primary outcome was polyp characterization.

Authors	Year	Recruitment	Machine learning approach	Image modality	Patients, n	Polypsor lesions, n	Total images, n	Images for training, n	Images for validation, n
Tischendorf et al [36]	2010	Prospective pilot	SVM^a^ classifier	Magnification NBI^b^	223	209	—^c^	208	—
Gross et al [37]	2011	Prospective	SVM classifier	Magnification NBI	214	434	—	433	—
Ganz et al [13]	2012	Retrospective	Shape-UCM^d^	NBI	—	—	—	58	87
Takemura et al [38]	2012	Retrospective	SVM classifier	Magnification NBI	—	371	—	1519	371
Mori et al [39]	2015	Retrospective	EC^e^-CAD^f^	EC	152	176	—	—	—
Kominami et al [11]	2016	Retrospective	SVM classifier	Magnification NBI	41	118	—	2247	—
Misawa et al [40]	2016	Retrospective	EndoBRAIN^g^	NBI and EC	—	85	1079	979	100
Mesejo et al [41]	2016	Retrospective	SfM^h^	White light and NBI	—	76	—	—	—
Mori et al [42]	2016	Retrospective	SVM classifier	EC-CAD	123	205	—	—	6051
Takeda et al [43]	2017	Retrospective	SVM classifier	EC-CAD	242	375	5843	5643	200
Byrne et al [44]	2017	Retrospective	DCNN^i^	NBI	—	125	—	60,089	—
Komeda et al [45]	2017	Retrospective	CNN	Endoscopic images	—	—	1200	—	—
Misawa et al [46]	2017	Retrospective	EndoBRAIN and ECV^j^-CAD	NBI	100	124	1834	173	1661
Mori et al [47]	2018	Retrospective	—	EC	—	144	—	—	—
Chen et al [48]	2018	Prospective	DNN^k^	NBI	193	284	2441	2157	284
Renner et al [49]	2018	Retrospective	DNN	NBI and HDWL^l^	250	231	788	602	186
Mori et al [50]	2018	Prospective	SVM classifier	NBI and EC	325	466	—	61,925	450
Mori et al [50]	2018	Prospective	SVM classifier	NBI and EC	325	466	—	61,925	450
Kudo et al [51]	2019	Retrospective	EndoBRAIN system	White light, NBI, and EC	89	100	—	69,142	5065
Kudo et al [51]	2019	Retrospective	EndoBRAIN system	White light, NBI, and EC	89	100	—	69,142	5065
Figueiredo et al [52]	2019	Retrospective	Segmentation algorithm	NBI	10	11	86	43	43
Rodriguez-Diaz et al [53]	2020	Retrospective	DeepLab framework	High magnification NBI	286	607	740	—	—
Yang et al [54]	2020	Retrospective	CNN-Inception-ResNet	White light	1339	—	3828	—	240
Zachariah et al [55]	2020	Retrospective	CNN-Inception-ResNet	NBI and white light	—	—	6223	—	634

^a^SVM: support vector machine.

^b^NBI: narrow band imaging.

^c^This value was not reported.

^d^Shape-UCM is an algorithm for automatic polyp segmentation.

^e^EC: endocytoscopy.

^f^CAD: computer-aided diagnosis.

^g^EndoBRAIN is a novel artificial intelligence system.

^h^SfM: structure from motion.

^i^DCNN: deep convolutional neural network.

^j^ECV: endocytoscopic vascular pattern.

^k^DNN: deep neural network.

^l^HDWL: high-definition white light.

**Table 3:** Characteristics of randomized controlled trials whose primary outcome was polyp detection.

Authors	Year	Recruitment	Machine learning approach	Imaging modality	Patients, n	Polyps, n	PDR^a^ –AI^b^, %	PDR –control, %	ADR^c^ –AI, %	ADR –control, %	Withdrawal time^d^; AI vs control, min	*P* value
Wang et al [56]	2019	Real-time, prospective	ANN^e^-SegNet architecture	Video stream	1058	767	45.02	29.10	29.12	20.34	6.18 vs 6.07	.15
Wang et al [57]	2020	Prospective	ANN-SegNet architecture	Video stream	962	809	52	37	34	28	6.48 vs 6.37	.14
Su et al [58]	2020	Prospective	DCNN^f^	Video stream	623	273	38.31	25.40	28.90	16.50	7.03 vs 5.68	<.001
Gong et al [59]	2020	Prospective	DCNN	Video stream	704	—^g^	47	34	16	8	6.38 vs 4.76	<.001
Liu et al [60]	2020	Prospective	ANN	Video stream	1026	734	43.65	27.81	39.10	23.89	6.82 vs 6.74	<.001
Luo et al [61]	2020	Prospective	CNN-YOLO^h^	Video stream	150	185	38.7	34.0	—	—	6.22 vs 6.17	.10
Repici et al [62]	2020	Prospective	CNN-GI Genius^i^	Video stream	685	596	—	—	54.8	40.4	6.95 vs 7.25	.10
Wang et al [63]	2020	Prospective	ANN-Endoscreener	Video stream	369	—	63.59	55.14	42.39	35.68	6.55 vs 6.51	.75

^a^PDR: polyp detection rate.

^b^AI: artificial intelligence.

^c^ADR: adenoma detection rate.

^d^Withdrawal time excluded the time to perform the biopsy.

^e^ANN: artificial neural network.

^f^DCNN: deep convolutional neural network.

^g^This value was not reported.

^h^YOLO: you only look once.

^i^GI Genius (Medtronic) is novel artificial intelligence system.

### Detection or Localization of a Polyp

**Figure 2:**
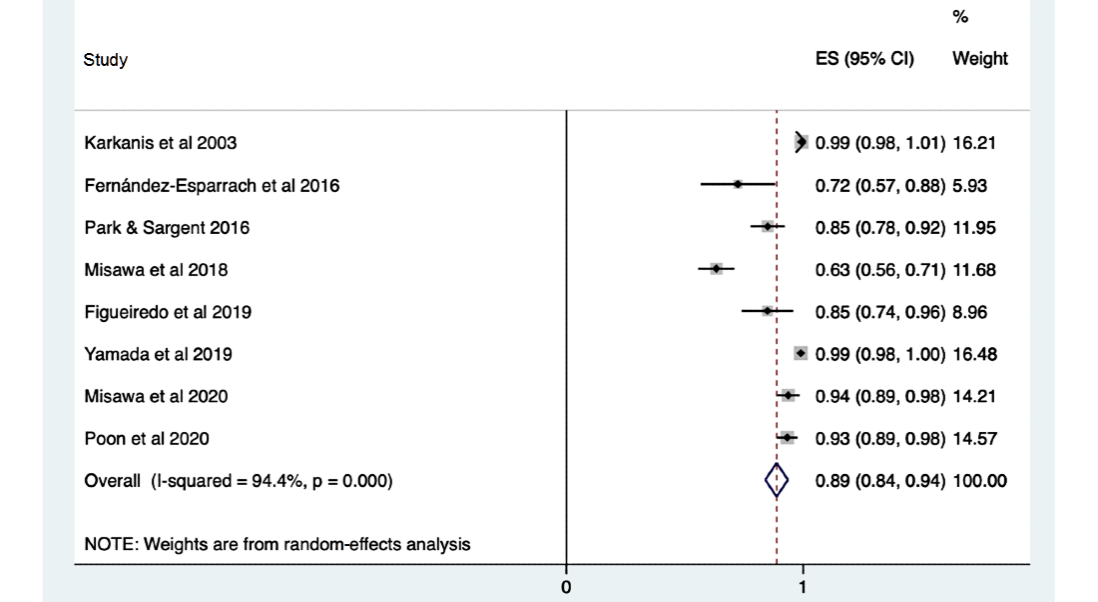
Pooled analysis of specificity of polyp detection by the use of lesions or polyps within video frames or images. Effect sizes (ES) are shown with 95% CIs. A random-effects model was used.

The diagnostic accuracy of the machine learning systems for detecting polyps was assessed using 103,049 still images in 10 studies, reporting a pooled sensitivity of 0.84 (95% CI 0.74-0.93), a specificity of 0.87 (95% CI 0.83-0.90), and an accuracy of 0.89 (95% CI 0.81-0.97). Lesions within video frames or images were used by 14 studies to report the diagnostic performance of their detection systems, highlighting a sensitivity of 0.92 (95% CI 0.89-0.95), a specificity of 0.89 (95% CI 0.84-0.94; Figure 2), and an accuracy of 0.87 (95% CI 0.76-0.97). There were 11 studies analyzing the accuracy of polyp detection through the use of images or video clips gathered from more than 17,401 patients. These demonstrated a sensitivity of 0.92 (95% CI 0.90-0.94), a specificity of 0.93 (95% CI 0.91-0.96), and accuracy of 0.92 (95% CI 0.87-0.98).

### Characterization of a Detected Polyp

**Figure 3:**
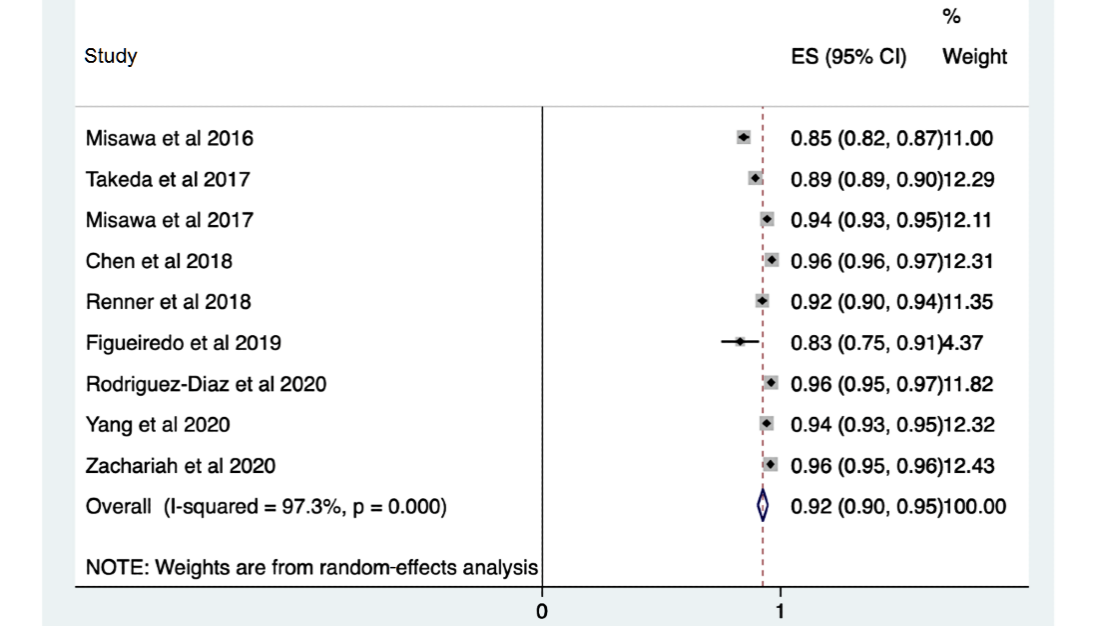
Pooled analysis of sensitivity of polyp characterization by the use of images. Effect sizes (ES) are shown with 95% CIs. A random-effects model was used.

There were 9 studies reporting diagnostic accuracy characteristics for computer analysis of single image frames. These included a total of 22,862 images and demonstrated a sensitivity of 0.92 (95% CI 0.90-0.95; Figure 3), a specificity of 0.79 (95% CI 0.68-0.91), and an accuracy of 0.87 (95% CI 0.83-0.91). A further 20 studies assessed the diagnostic accuracy of techniques for predicting the histological diagnosis of a polyp, with a sensitivity of 0.94 (95% CI 0.92-0.95), a specificity of 0.87 (95% CI 0.83-0.90), and an accuracy of 0.91 (95% CI 0.88-0.93). A total of 16 studies analyzed diagnostic accuracy using images or video clips from a cohort of 4001 patients having undergone colonoscopy. These studies showed a sensitivity of 0.94 (95% CI 0.92-0.95), a specificity of 0.82 (95% CI 0.73-0.91), and an accuracy of 0.90 (95% CI 0.86-0.94).

### PDR and ADR for Polyp Detection: RCTs

The 8 RCTs consisted of a total of 5577 patients: 2438 patients in the AI group and 2463 patients in the control group with standard colonoscopy alone [56-59]. These captured data prospectively with the use of deep learning methods on real-time video streams from colonoscopy.

**Figure 4:**
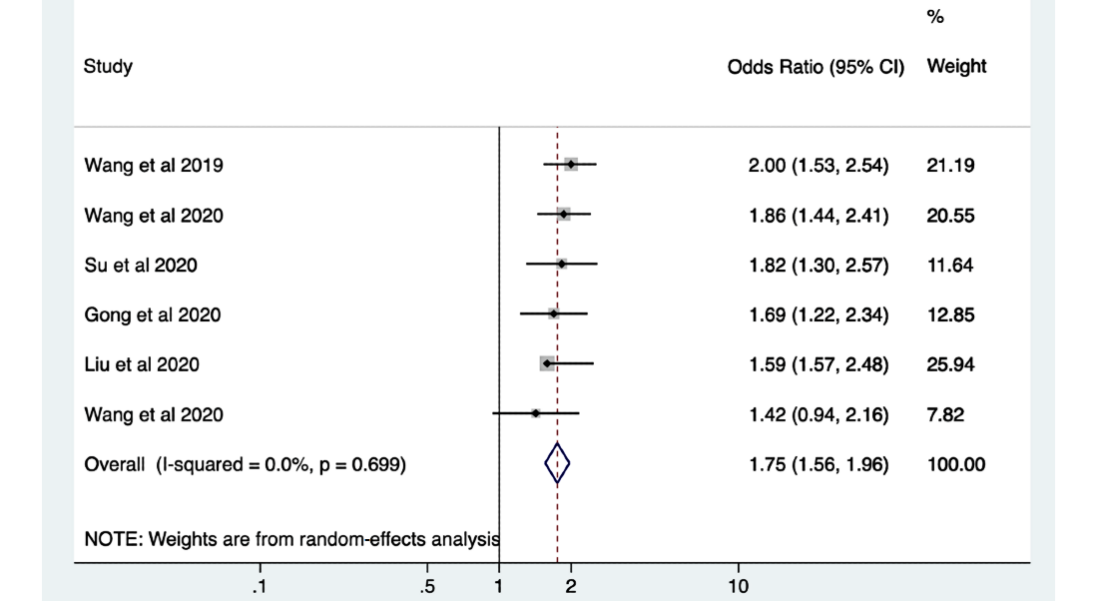
Pooled analysis of polyp detection rate. Odds ratios are shown with 95% CIs. A random-effects model was used for the meta-analysis.

**Figure 5:**
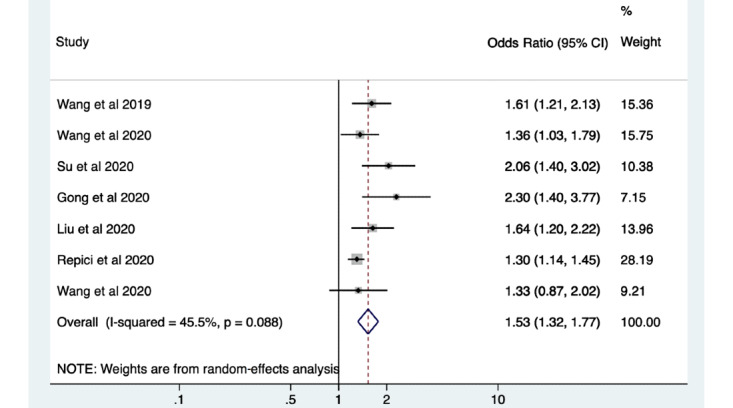
Pooled analysis of adenoma detection rate. Odds ratios are shown with 95% CIs. A random-effects model was used for the meta-analysis.

The meta-analysis showed a significant increase in pooled PDR in patients with the use of AI for polyp detection during colonoscopy compared with patients who had standard colonoscopy (odds ratio [OR] 1.75, 95% CI 1.56-1.96; *P*<.001; Figure 4). The PDR ranged from 38% to 64% when using AI, with a median of 45%. When comparing patients undergoing colonoscopy with the use of AI to those having standard colonoscopy, there was also a significant increase in ADR (OR 1.53, 95% CI 1.32-1.77; *P*<.001; Figure 5). The ADR ranged from 16% to 55% with a median of 34% when using AI technology compared to standard colonoscopy.

### Heterogeneity of Studies

There was a high degree of variation between studies. The heterogeneity was statistically significant when comparing the studies for polyp detection and characterization and assessing for sensitivity, specificity, and accuracy (*P*<.05). The lowest variation for polyp detection was among the studies assessing accuracy with polyp data (I^2^=86.3%), and the highest was among those analyzing the sensitivity of machine learning systems using image data sets (I^2^=99.9%). When considering studies for polyp characterization, the heterogeneity was lowest for studies analyzing sensitivity using patient data sets (I^2^=51.1%) and highest when assessing specificity using image data sets (I^2^=99.9%). Within the RCTs assessed, there was found to be a low degree of heterogeneity for PDR (I^2^=0%; *P*=.70) and a moderate degree of heterogeneity for ADR (I^2^=45.5%; *P*=.09). These results were not statistically significant.

### Quality Assessment

The assessment of bias for the studies when using the QUADAS-2 tool is depicted in Table S1 in Multimedia Appendix 2. Most of the RCTs scored 3 or more on the Jadad scale and were, therefore, considered to be of good quality (Table S2 in Multimedia Appendix 2). One study scored 2, suggesting poor quality, but after reviewing the paper and its evidence in detail, the paper was included in the final analysis [64]. This is because despite the lack of mention of blinding, the selection process for participants was justified with consecutive patients enrolled, and there were no concerns regarding applicability. The paper matched the selection criteria of our study and was otherwise in line with other studies that were included.

## Discussion

### Principal Findings

The aim of this systematic review and meta-analysis was to examine the current status of diagnostic accuracy for AI-based technologies in the detection and characterization of colorectal polyps. We found a wide variety of machine learning systems being used for polyp detection and characterization in numerous studies. The overall diagnostic accuracy for these systems to detect polyps was high, predominantly with sensitivities, specificities, and accuracies above 84%. When characterizing polyps, the majority of machine learning systems had sensitivities, specificities, and accuracies above 82%. These outcomes show good results for current machine learning systems and algorithms to detect and characterize polyps, and indirectly in regard to the rate of false positives.

This meta-analysis highlights a significant increase in PDR and ADR when using AI systems in conjunction with colonoscopy in real time to detect polyps in the colon and rectum with an overall OR of 1.75 (95% CI 1.56-1.96; *P*<.05) and 1.53 (95% CI 1.32-1.77; *P*<.05), respectively. The UK key performance indicators and quality assurance standards for colonoscopy dictate that the minimal ADR should be 15%, with an aspirational target of 20% [65]. It has previously been shown that endoscopists with an ADR of less than 20% had a hazard ratio for interval cancer that was 10 times higher than those with an ADR of greater than 20% [66]. All RCTs in this review were shown to have an ADR of greater than 15% when detecting polyps with the use of an AI system, the majority of which highlighted an ADR of greater than 25% [56-58]. These outcomes are a promising start for the use of AI to detect missed polyps and, thus, may lead to a reduction in CRC incidence.

The assessment of quality of the diagnostic accuracy studies included in this paper highlighted an overall low risk of bias, justifying the validity of the study results and implying that their results may be applicable to clinical practice. The main area of bias in the RCTs was in the process of blinding. This may have contributed to an overestimation in the effects of AI in polyp detection.

There are many limitations within the published studies (Table S1 in Multimedia Appendix 3). Factors contributing to the miss rate of polyps are multifactorial and include patient-related factors, polyp-related factors, and image-related factors [67,68]. It is encouraging to note that a variety of imaging modalities were used in the studies in this review, since this will improve applicability in a clinical setting. We note that most studies with image enhancement techniques have used NBI, and it will be important to validate the performance of AI systems in endoscopy using image enhancement approaches from other manufacturers (eg, i-scan from PENTAX Medical and blue laser imaging from Fujifilm Corporation). Some studies analyzing polyp characterization used magnification NBI [11,36,38,69]. This imaging modality is not commonly used in Western endoscopic practice, so is less applicable to a health care setting in the Western world. Although there has been significant development in computer-assisted technologies to increase ADR, issues with image quality still remain. Many studies in this review excluded images that were blurred or of poor quality when assessing diagnostic accuracy of the machine learning systems. [27,40,42,51]. Recent RCTs have tried to tackle this problem by developing models to recognize blurry frames [58,59]. Other studies excluded images with poor bowel preparation [27,36,48]. Adequate bowel cleansing is vital for complete mucosal inspection; however, it has been shown in a meta-analysis that low-quality preparation does not significantly affect ADR, since these patients frequently undergo repeat colonoscopy [70]. Most RCTs included in this review used the Boston Bowel Preparation Scale [71] to assess adequacy of bowel preparation.

Sufficient withdrawal time allows full mucosal inspection with careful examination of all folds and flexures, in an attempt to avoid missing any polyps. It has been shown that an increase in withdrawal time is associated with an increase in ADR [72]. This supports the use of withdrawal times as a quality indicator for screening colonoscopy. In preclinical studies, it is difficult to assess withdrawal times given the use of still images and video clips. In the RCTs assessed, the withdrawal times—excluding biopsy time—were mostly higher with the use of AI-based technology, although not significantly so in all studies (Table 3). However, the ability to record the withdrawal time is equally important [58,59]. This may suggest that quality control during colonoscopy examinations can be maintained with the use of machine learning.

Given the fact that AI is a relatively new and evolving area of medical practice, there is a lack of evidence-based standards to support its development. This is highlighted through the inconsistencies in validating the machine learning systems in each study. The data used for training the algorithms vary in type, for example, as either a static image from the colonoscopy [45,46] or an image of a polyp [21,47], and in number, with some studies having very small sample sizes [21,52]. We acknowledge the high degree of heterogeneity in the included studies, which may, in part, be explained by the wide range of approaches or algorithms used. This may suggest that our findings are applicable to a wide range of study settings and outcomes. However, the high degree of heterogeneity also emphasizes the issue of inconsistencies within the development of AI systems and, thus, weakens their design and may hinder implementation of the AI systems in a clinical setting. In order to address this problem, we are developing a new multidisciplinary, consensus-based reporting standards statement called STARD-AI (Standards for Reporting of Diagnostic Accuracy Studies–Artificial Intelligence). It is being developed to provide stringent guidelines for all AI-based clinical trials that report diagnostic accuracy [73,74].

The lack of standards among these studies introduces an element of selection bias. In traditional computer programming, intelligent systems were built by writing models by hand and, therefore, understanding the rules from which conclusions were made. Neural networks and deep learning techniques are criticized for their “black box” problem, in failing to produce an intelligible description of the results produced. This creates tension between our need for explanations and our interests in efficiency. Most studies in this systematic review did not reveal their algorithms, which begs one to question whether they only used the algorithms that were most successful in producing the desired outcome without understanding the process underlying it.

Multiple other factors contribute to the lack of applicability of these studies in clinical practice. Many of the studies about polyp detection and characterization have been carried out in Japan [46,50,51] or China [19,56,59], and differences in polyp biology and tumorigenesis may limit application to Western endoscopy practice [75]. Furthermore, for real-time detection to be successful, the operation of the AI system to detect and characterize polyps must be fast, practical, and nondisruptive to workflow. However, most current studies are designed in a nonclinical environment and carried out retrospectively, with only a handful of recent RCTs. More RCTs are needed to provide prospective data by testing the machine learning systems while a colonoscopy procedure is undertaken.

The financial implications of introducing an AI system to endoscopy should be considered. The studies in this review lack evidence to show that AI systems would be cost-effective. Before clinical application, studies must demonstrate that the current burden on health care systems and histopathology departments can be relieved, both in view of workload and in terms of costs. A very recent study examining the use of AI combined with the diagnose-and-leave strategy for diminutive polyps has found substantial reductions in the cost of colonoscopy based on prospective data [76]. This is an encouraging outcome, but more studies are needed.

The role of the health care workforce must also be considered in a time of developing AI systems. At present, real-time detection systems during colonoscopy are not able to operate independently of human direction, but understanding the change in the role of the endoscopist and nurses will be crucial for the future. In addition, a skills gap to prepare the workforce for AI will need to be addressed. The refinement of machine learning systems in detecting polyps will eventually lead to the use of AI in conjunction with all routine colonoscopy procedures. This will allow the procedure to be performed by staff who will not require the lengthy training or accreditation [77]. In this scenario, only patients with complex polyps requiring more advanced management may need to be referred to expert endoscopists.

It is important to also consider some of the ethical dilemmas that arise from the use of AI in health care. The aim of AI in polyp detection and characterization is to introduce machine learning as a “checker system” for the endoscopist. As a result, incorporation of AI into endoscopy should be encouraged as a complementary tool and not as a replacement for a clinician. For this reason, a high degree of accuracy is required from AI systems. We expect that they operate with 100% sensitivity and a low rate of false positives. However, AI is not yet free from bias or errors, and an AI decision support tool could easily succumb to automation bias when its predictions are almost always followed by the endoscopist [78]. Machine learning systems can also unintentionally reproduce or magnify existing biases of their training data sets and exacerbate health disparities [79]. Many of the studies in this meta-analysis, for example, have excluded patients with IBD or sessile serrated polyps [39,43,56], limiting their applicability for these populations. We recognize that these other cohorts of patients, including those with benign colonic pathologies and not exclusively polyps, are important to include in such research. However, this technology is still in its infancy and these patient groups represent a minority. It is difficult and not entirely feasible to create validated AI algorithms for all patient cohorts until the technology is more established and works well in its own right.

Although this systematic review has shown the performance of the AI systems to be satisfactory, the majority of the studies are preclinical trials that have not addressed these clinical needs. As a result, there remains a lack of confidence by endoscopists and patients to fully adopt the system as a whole. The clinical expectations exceed the aims of the machine learning algorithms. To fully support the incorporation of an AI system into routine practice, the diagnostic accuracy for polyp detection and characterization must meet the desired threshold, while also providing confidence that quality requirements will be fulfilled.

A further two challenges threaten the ability for AI to thrive in health care: patient confidentiality and accountability. The lack of stringent policies for the use of training data in AI means that the methods used to deidentify patient information are weak, and we suggest that standardized guidance is required for the consent of collection and use of patient data for AI training purposes. Once an algorithm-based health care system is operational, the question of accountability arises. In the case that a machine learning system working in unison with an endoscopist detects and characterizes a polyp as hyperplastic when, in fact, it is adenomatous, who is held liable for this mistake? A robust legal framework in association with national and international endoscopy representative groups (eg, the Joint Advisory Group on Gastrointestinal Endoscopy in the United Kingdom and the ASGE in the United States) for the use of AI in health care is vital to protect endoscopists and patients. Addressing these important concerns will help build confidence and trust among patients and doctors for the use of machine learning in the delivery of care.

### Conclusions

This systematic review and meta-analysis highlights the growing interest in the field of polyp detection and characterization during colonoscopy using AI. The current accuracy of machine learning for this role is high. There is potential to improve ADR and, consequently, reduce the incidence of CRC.

However, AI and machine learning systems are still evolving. Firstly, higher-quality research with modern trial designs is needed in this field, with particular attention on using larger data sets and by validating the AI systems prospectively in a clinical setting. Secondly, these systems must provide quality assurance with a robust ethical and legal framework before they can be fully embraced by clinicians and patients in the future.

## Appendix

Multimedia Appendix 1 Search strategy for studies to include.

Multimedia Appendix 2 Quality assessment of the studies.

Multimedia Appendix 3 Limitations within the published studies.

## Abbreviations

ADR: adenoma detection rate
AI: artificial intelligence
ASGE: American Society of Gastrointestinal Endoscopy
CAD: computer-aided diagnosis
CNN: convolutional neural network
CRC: colorectal cancer
DCNN: deep convolutional neural network
IBD: inflammatory bowel disease
NBI: narrow band imaging
OR: odds ratio
PDR: polyp detection rate
PIVI: Preservation and Incorporation of Valuable endoscopic Innovations
PRISMA: Preferred Reporting Items for Systematic Reviews and Meta-Analyses
PROSPERO: International Prospective Register of Systematic Reviews
QUADAS-2: Quality Assessment of Diagnostic Accuracy Studies 2
RCT: randomized controlled trial
STARD-AI: Standards for Reporting of Diagnostic Accuracy Studies–Artificial Intelligence
