# Supplementary material for: Diagnostic Accuracy of Artificial Intelligence and Computer-Aided Diagnosis for the Detection and Characterization of Colorectal Polyps: Systematic Review and Meta-analysis
Source: J Med Internet Res. 2021 Jul 14;23(7):e27370. doi: 10.2196/27370 (PMC8319784; doi:10.2196/27370)
Supplement: Multimedia Appendix 1 [file jmir_v23i7e27370_app1.docx]

**Multimedia Appendix 1 - Search Strategy**

EMBASE & Medline

#1 Colonic Polyps/

#2 colorect* or colon or colonoscop* or large bowel or rectal or rectum or anal or anus or large intestine and polyp

#3 1 or 2

#4 exp algorithms/

#5 artificial intelligence or machine learning or neural network*

#6 4 or 5

#7 computer aided or computer assisted and diagnos* or detect*

#8 diagnosis, computer-assisted/ or image interpretation, computer-assisted/ or neuronavigation/

#9 image processing, computer-assisted/ or data compression/ or image enhancement/ or imaging, three-dimensional/

#10 7 or 8 or 9

#11 3 and 6 and 10

The Cochrane Library

#1 Colonic Polyps

#2 colorect* or colon or colonoscop* or large bowel and polyp

#3 1 or 2

#4 exp artificial intelligence

#5 artificial intelligence or machine learning or neural network*

#6 4 or 5

#7 exp diagnosis, computer-assisted

#8 computer aided or computer assisted and diagnos* or detect*

#9 7 or 8

#10 3 or 6 or 9
