# Supplementary material for: Diagnostic Accuracy of Artificial Intelligence and Computer-Aided Diagnosis for the Detection and Characterization of Colorectal Polyps: Systematic Review and Meta-analysis
Source: J Med Internet Res. 2021 Jul 14;23(7):e27370. doi: 10.2196/27370 (PMC8319784; doi:10.2196/27370)
Supplement: Multimedia Appendix 2 [file jmir_v23i7e27370_app2.docx]

**Multimedia Appendix 2 – Quality Assessment**

**Table S1.**Assessment of bias for each included study in seven domains according to the QUADAS-2 tool. ☺ = low risk of bias; ☹ = high risk of bias; ? = unclear risk of bias.

| **Study** | **RISK OF BIAS** | | | | **APPLICABILITY CONCERNS** | | |
| --- | --- | --- | --- | --- | --- | --- | --- |
|  | **PATIENT SELECTION** | **INDEX TEST** | **REFERENCE STANDARD** | **FLOW AND TIMING** | **PATIENT SELECTION** | **INDEX TEST** | **REFERENCE STANDARD** |
| **Detection** |  | | | | | | |
| Karkanis et al. 2003 | ☹ | ☺ | ☺ | ☺ | ? | ☺ | ☺ |
| Fu et al. 2014 | ☺ | ? | ☺ | ? | ☺ | ☺ | ☺ |
| Wang et al. 2015 | ☹ | ☹ | ☺ | ? | ? | ? | ☺ |
| Tajbakhsh et al. 2015 (a) | ☺ | ☺ | ☹ | ? | ☺ | ☺ | ☺ |
| Tajbakhsh et al. 2015 (b) | ☺ | ☺ | ☹ | ? | ☺ | ☺ | ☺ |
| Fernández-Esparrach et al. 2016 | ? | ? | ☺ | ? | ? | ☺ | ? |
| Park & Sargent 2016 | ☹ | ☺ | ☹ | ☺ | ☹ | ☺ | ? |
| Urban et al. 2018 | ☺ | ☺ | ☺ | ? | ☹ | ☺ | ☺ |
| Wang et al. 2018 | ☺ | ☺ | ☺ | ☺ | ☺ | ☺ | ☺ |
| Misawa et al. 2018 | ☺ | ☺ | ? | ☺ | ☺ | ☺ | ? |
| Figueiredo et al. 2019 | ☹ | ☺ | ? | ☺ | ? | ☺ | ☺ |
| Yamada et al. 2019 | ☹ | ☺ | ? | ☺ | ? | ☺ | ☺ |
| Becq et al. 2020 | ☺ | ☺ | ☺ | ? | ☺ | ☹ | ☺ |
| Gao et al. 2020 | ☹ | ☺ | ☹ | ? | ☺ | ☺ | ☹ |
| Guo et al. 2020 | ☹ | ☺ | ☺ | ☺ | ? | ☺ | ☺ |
| Lee et al. 2020 | ☺ | ☺ | ☺ | ? | ☺ | ☺ | ☺ |
| Ozawa et al. 2020 | ? | ☺ | ☺ | ☺ | ☹ | ☺ | ☺ |
| Misawa et al. 2020 | ☺ | ☺ | ☺ | ☺ | ☺ | ☺ | ☺ |
| Poon et al. 2020 | ☺ | ☺ | ? | ? | ? | ☺ | ☺ |
| **Characterisation** |  | | | | | | |
| Tischendorf et al. 2010 | ☺ | ☺ | ☺ | ☺ | ☺ | ☺ | ☺ |
| Gross et al. 2011 | ☺ | ☺ | ☺ | ☺ | ☺ | ☺ | ☺ |
| Ganz et al. 2012 | ☺ | ☺ | ☹ | ? | ☹ | ☺ | ? |
| Takemura et al. 2012 | ☹ | ☺ | ☺ | ? | ? | ☺ | ☺ |
| Mori et al. 2015 | ☹ | ☺ | ☺ | ☺ | ☺ | ☺ | ☺ |
| Kominami et al. 2016 | ? | ☺ | ? | ☺ | ? | ☺ | ? |
| Misawa et al. 2016 | ☺ | ☺ | ? | ? | ? | ☺ | ? |
| Mesejo et al. 2016 | ? | ? | ? | ☺ | ? | ☺ | ☺ |
| Mori et al. 2016 | ☹ | ☺ | ? | ☺ | ☹ | ☺ | ☺ |
| Takeda et al. 2017 | ☺ | ☺ | ☹ | ☺ | ☺ | ☺ | ? |
| Byrne et al. 2017 | ? | ☺ | ☹ | ? | ☹ | ☺ | ? |
| Komeda et al. 2017 | ☹ | ☺ | ☺ | ☺ | ☹ | ☺ | ☺ |
| Misawa et al. 2017 | ☺ | ☺ | ☺ | ? | ☺ | ☺ | ☺ |
| Mori et al. 2018 | ☹ | ☺ | ☺ | ? | ☹ | ? | ☺ |
| Chen et al. 2018 | ? | ☺ | ☺ | ☺ | ☺ | ☺ | ☺ |
| Renner et al. 2018 | ☺ | ☺ | ☺ | ☺ | ☺ | ☺ | ☺ |
| Mori et al. 2018 (a) | ☺ | ☺ | ☺ | ☹ | ☺ | ☺ | ☺ |
| Mori et al. 2018 (b) | ☺ | ☺ | ☺ | ☹ | ☺ | ☺ | ☺ |
| Kudo et al. 2019 (a) | ? | ☺ | ☺ | ☺ | ☺ | ☺ | ☺ |
| Kudo et al. 2019 (b) | ? | ☺ | ☺ | ☺ | ☺ | ☺ | ☺ |
| Figueiredo et al. 2019 | ? | ☺ | ? | ☺ | ☹ | ☺ | ? |
| Rodriguez-Diaz et al. 2020 | ? | ☺ | ☹ | ? | ☺ | ☺ | ☹ |
| Yang et al. 2020 | ☺ | ☺ | ☺ | ☺ | ☺ | ☺ | ☺ |
| Zachariah et al. 2020 | ☹ | ☺ | ☺ | ? | ☹ | ☺ | ☺ |

**Table S2.** Assessment of bias for RCTs using the Jadad scale; score ≥ 3 = good quality.

| **Study** | **Randomisation (2)** | **Blinding (2)** | **An account of all patients (1)** | **Total / 5** |
| --- | --- | --- | --- | --- |
| Wang et al. 2019 | 2 | 1 | 1 | **4** |
| Wang et al. 2020 | 2 | 2 | 1 | **5** |
| Su et al. 2020 | 2 | 2 | 1 | **5** |
| Gong et al. 2020 | 2 | 2 | 1 | **5** |
| Liu et al. 2020 | 1 | 0 | 1 | **2** |
| Luo et al. 2020 | 2 | 0 | 1 | **3** |
| Repici et al. 2020 | 2 | 1 | 1 | **4** |
| Wang et al. 2020 | 2 | 2 | 1 | **5** |
