# Supplementary material for: Diagnostic Accuracy of Artificial Intelligence and Computer-Aided Diagnosis for the Detection and Characterization of Colorectal Polyps: Systematic Review and Meta-analysis
Source: J Med Internet Res. 2021 Jul 14;23(7):e27370. doi: 10.2196/27370 (PMC8319784; doi:10.2196/27370)
Supplement: Multimedia Appendix 3 [file jmir_v23i7e27370_app3.docx]

**Multimedia Appendix 3 - Limitations**

**Table S1.** Limitations of current studies assessing polyp detection or characterisation.

| **Factors** | **Variables** |
| --- | --- |
| **Study design** | Retrospective or prospective data collection |
| **Datasets** | Image, polyp or patient |
| **Size of datasets** | Wide variation in size of datasets for testing and validating |
| **Imaging modality** | Different imaging modalities used e.g. white light, NBI, EC |
| **Machine learning approach** | Variety in type of algorithm used |
| **Population** | Eastern vs. Western population |
| **Reporting of outcome** | Different diagnostic accuracy metric reported - sensitivity, specificity, accuracy, ADR, PDR |
| **Applicability** | Pre-clinical studies vs. clinical trials |
